# Can oral minoxidil be the game changer in androgenetic alopecia? A comprehensive review and meta-analysis comparing topical and oral minoxidil for treating androgenetic alopecia

**DOI:** 10.1093/skinhd/vzaf009

**Published:** 2025-04-25

**Authors:** Faizan Fazal, Bilal Haider Malik, Haris Mumtaz Malik, Beenish Sabir, Haris Mustafa, Mushood Ahmed, Areesha Abid, Maham Leeza Adil, Umar Shafi, Muhammad Saad

**Affiliations:** Department of Medicine, Holy Family Hospital, Rawalpindi, Pakistan; Department of Dermatology, Betsi Cadwaladr University Health Board, Wrexham Maelor Hospital, Wrexham, UK; Department of Medicine, Holy Family Hospital, Rawalpindi, Pakistan; Department of Medicine, Holy Family Hospital, Rawalpindi, Pakistan; Department of Medicine, Holy Family Hospital, Rawalpindi, Pakistan; Department of Medicine, Holy Family Hospital, Rawalpindi, Pakistan; Department of Medicine, Holy Family Hospital, Rawalpindi, Pakistan; Department of Geriatrics, Albert Edward Infirmary Hospital, Wigan, UK; Department of Medicine, Holy Family Hospital, Rawalpindi, Pakistan; Department of Medicine, Holy Family Hospital, Rawalpindi, Pakistan

## Abstract

Androgenetic alopecia (AGA) is a hair disorder seen in both sexes. Its aetiology is multifactorial. Treating AGA has always been a challenge for dermatologists. Only a few drugs such as topical minoxidil and finasteride are U.S. Food and Drug Administration-approved for treating AGA. Thus, looking for new and more effective treatment options for AGA is imperative. This review was conducted to compare the efficacy of oral with topical minoxidil in treating AGA. Only clinical trials that compared oral with topical minoxidil in treating AGA were included in this review. PubMed, Cochrane, Scopus and ClinicalTrials.gov were searched. A total of 2063 studies were retrieved from the databases. Four studies met the inclusion criteria and were included in this systematic review. Outcomes such as hair density, terminal hair density, hair count, global photographic assessment and negative hair pull were studied in this review. The results showed that there was no significant difference between oral and topical minoxidil in terms of improving the hair density of patients with AGA (overall mean difference 0.95, 95% confidence interval −24.98 to 26.87). Two studies showed that the mean difference in terminal hair density was greater in patients treated with oral minoxidil, but there was no significant difference between the two treatments in terms of improving terminal hair density. The efficacy of oral minoxidil is comparable to that of topical minoxidil in terms of improving hair density and terminal hair density in patients with AGA. Oral minoxidil can be used as an adjunct or as a second-line treatment option for AGA.

## Teaser

The efficacy of oral minoxidil is comparable to topical minoxidil in terms of improving hair density and terminal hair density in AGA patients. Oral minoxidil can be used as an adjunct or as a second-line treatment option for AGA.

The most prevalent nonscarring alopecia is androgenetic alopecia (AGA), which is marked by a distinct, gradually patterned hair loss. Genetic susceptibility and increased follicular sensitivity to androgens induce AGA. It is relatively common and can have serious psychological effects.^1^ AGA is not only associated with ageing and heredity, but is also influenced by other variables, such as lifestyle.^2^

Research indicates that AGA is associated with a high prevalence of endocrine disorders and other comorbidities. It could be a skin ailment or an early sign of an underlying systemic disorder.^3^ All populations are seeing an increase in the disease’s frequency, age of onset and severity. The primary physiological depiction of AGA is the continual reduction in size of terminal hair follicles, which ultimately results in their conversion into vellus hair or atrophy.^4^ This can manifest in men as balding, a receding frontal hairline and thinning hair over the vertex scalp. In women, it usually manifests as thinning hair across the vertex of the scalp. Treatments include topical minoxidil (TM), hormone therapy, 5 alpha-reductase inhibitors and hair transplantation, but the effectiveness of these treatments varies greatly.^5^

AGA is often diagnosed by looking at the clinical symptoms and the patient’s history. The doctor or other healthcare provider should review the patient’s medication regimen, gynaecological history for women, baseline blood tests (such as thyroid stimulating hormone, complete blood count, iron, ferritin and vitamin D), and medical and family history to rule out other causes of hair loss.^6^

It is still challenging to choose a treatment for AGA because it requires ethical, evidence-based decision-making that takes into account the needs, compliance, finances, degree of hair loss and aesthetic goals of each patient, regardless of any potential financial rewards for the practitioners. The choice of effective therapy for this chronic illness might be difficult.^7^ The U.S. Food and Drug Administration (FDA) has only approved TM and finasteride as medicinal therapies. The only device for treating AGA that has received FDA approval is the Lasermax haircomb.^8^ Oral minoxidil (OM) was first prescribed as an antihypertensive drug in the 1970s. It is currently used for several hair loss disorders globally. It is used off-license for several other hair loss issues.^9^

Researchers have shown that minoxidil lengthens the anagen phase of dermal papilla cells by increasing prostaglandin E2 production and vascular endothelial growth factor in a dose-dependent way.^10^ For individuals aged > 18 years, TM is available in the form of 2% and 5% formulations. For men, the 5% formulation is preferable. Six months should pass before patients are evaluated.^9^ According to studies, at 6 months, 1 year and 5 years, both the 2% and 5% formulations of TM significantly outperformed placebo in terms of hair growth. The 2% and 5% TM formulations demonstrated encouraging gains in female pattern hair loss in women.^11^

Recently, there has been increased interest in using low-dose OM to treat AGA, although the use of OM in treating AGA is not yet FDA approved. The dosage of OM can be customized by splitting a 2.5-mg tablet in half or quarters, according to the preferences of the patient and the healthcare professional.^9^ When Ramos *et al*. evaluated the effectiveness of a TM 5% solution daily vs. OM 1 mg daily, they discovered that TM was not more effective than OM. Furthermore, there was a greater improvement in hair loss ratings while using OM.^12^ This review aimed to compare OM with TM in improving outcomes in patients with AGA.

## Methods

### Type of study

We carried out a systematic review and meta-analysis of clinical trials comparing OM with TM in the treatment of patients with AGA.

### Eligibility criteria

Men and women with AGA were included in this review. Double-arm clinical trials that compared OM with TM in treating AGA were included. Forms of alopecia other than AGA were excluded from the study. Studies other than clinical trials were also excluded.

### Search strategy

Databases including PubMed, Cochrane, Scopus and ClinicalTrials.gov were searched using the key terms ‘Minoxidil’ and ‘Alopecia’ to find and shortlist the relevant studies.

### Statistical methods

Comprehensive Meta-Analysis V3 (https://meta-analysis.com/) was used for the statistical analysis. A forest plot was constructed showing a comparison between OM and TM for hair density outcome, along with a 95% confidence interval (CI). CIs were computed from *P*-values using the formula z = −0.862 + √[0.743 – 2.404 × log(*P*-value)] following the natural log transformation of the estimate, if not already provided in the study. Heterogeneity was evaluated using *I*^2^.

### Statistical analysis aims

A forest plot was constructed with RevMan (https://revman.cochrane.org/info) to study the outcome of hair density in OM and TM groups. Heterogeneity and *P*-values were calculated to assess the significance of the results.

### Risk of bias

Risk of bias graphs were made with the RoB 2 tool (https://www.riskofbias.info/welcome/rob-2-0-tool). Quality assessment was performed to evaluate the risk of bias in the studies included. The bias evaluation was carried out by two authors individually with the RoB 2.0 tool for systematic intervention reviews. This assessment considers multiple domains of bias, including those related to randomization, selective reporting, missing outcomes data, intervention adherence and outcome measurement

### Assumptions

Whether OM is better than TM in improving the outcomes of patients with AGA was checked.

### Outcomes

The studied outcomes were hair density, terminal hair density, hair count, global photographic assessment and negative hair pull.

### Data screening and data extraction

Search results were imported into Rayyan (https://www.rayyan.ai/). Using Rayyan, two investigators (H.M.M. and F.F.) independently screened the title and abstract of each reference for inclusion. The full text of potentially relevant articles was assessed independently by two reviewers (A.A. and H.M.). Discrepancies were resolved by discussion with the entire group.

Data extraction was conducted rigorously and transparently by two authors independently, following predefined inclusion criteria and using a standardized data-extraction sheet. Any discrepancies or differences of opinion during the data extraction process were resolved through discussion and consensus between the authors, with the involvement of a third reviewer or author if necessary. Two investigators independently screened each citation for inclusion. To ascertain their eligibility, two reviewers (B.H.M. and M.A.) independently examined the full-text publications of possibly pertinent studies. The following data were extracted from the included studies: study name, year, URL/DOI, study type, sample size, inclusion and exclusion criteria, intervention, comparator, follow-up, outcome, adverse events, potentially poolable outcome, the baseline characteristics included, study ID, year of publication, study design, sample size, outcomes assessed and results.

### Handling of missing data

All included articles were searched for all the included outcomes. Missing data are clearly mentioned in the tables presented in the ‘Results’ section. Data related to the outcome of hair density were available in three of the included articles. However, data were not available for the remaining outcomes in the majority of the included articles. This is also highlighted in the article when the outcomes are being discussed. More randomized controlled trials (RCTs) are required on this topic to cover and give insight into those specific outcomes for which data are not uniformly available. We have kept in mind the unavailability of data related to certain outcomes and have given our opinion in the ‘Discussion’ accordingly.

### Original data access

The data for all four included studies are available in full-length versions of each of the four articles. The extracted original data of all the included studies are available via the URL provided in the ‘Data availability’ statement.

## Results

**Figure 1:**
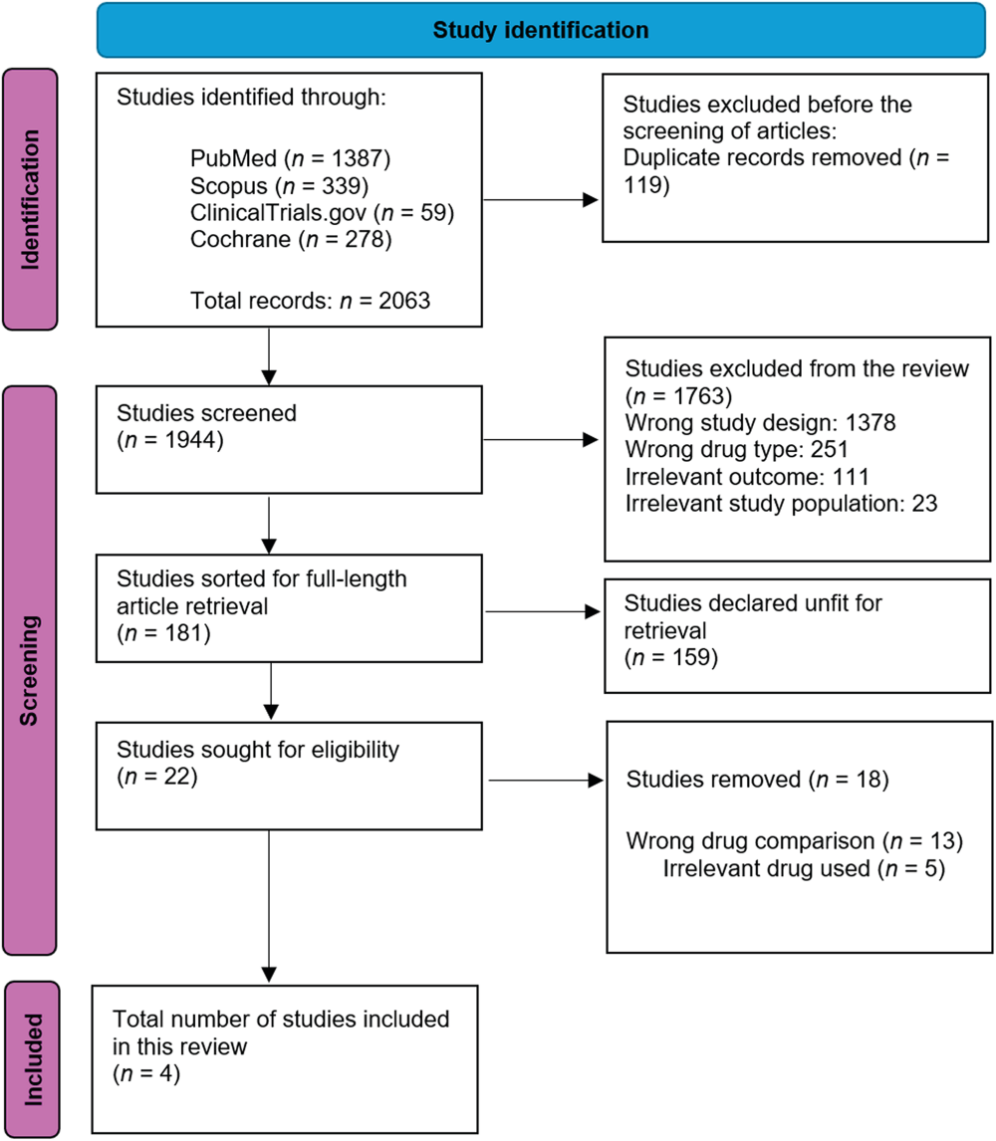
PRISMA flowchart showing study selection scheme.

Out of a total of 2063 studies, only 4 were considered to meet our inclusion criteria and were included in this systematic review.^12–15^ The study selection is shown in Figure 1.

### Features of the included studies

All four of the included studies were RCTs. A total of 257 patients with AGA were studied. These patients were randomized into OM and TM groups. The OM dosage administered to patients in each study was 1 mg once daily. All studies used TM 5%, except for that of Vahabi-Amlashi et al.,^14^ where patients were given TM 2%. Details of the studies that were included in this review are provided in Table 1.

**Table 1:** Features of the studies that were included in this review

Study feature	Ramos et al.^12^	Asilian et al.^13^	Vahabi-Amlashi et al.^14^	Penha 2024^15^
Country	Brazil	Iran	Iran	Brazil
Study design	RCT	RCT	RCT	RCT
Patients (*n*)	52	65	72	68
Participant age range (years)	18–65	18–56	18–50	18–55
Sex	Female	Male and female	Female	Male
No. of patients in OM group	26	33	36	33
OM dosage	1 mg OM once daily for 24 weeks	1 mg OM once daily for 24 weeks	1 mg OM once daily for 9 months	1 mg OM once daily for 24 weeks
No. of patients in TM group	26	29	33	33
TM dosage	TM 5% solution 1 mL once daily for 6 months	TM 5% solution 1 mL once daily for women and twice daily for men for 6 months	TM 2% solution 1 mL once daily for 9 months	TM 5% solution 1 mL twice daily for 6 months
Primary outcome	Change in total hair density	Hair thickness, hair count, and photographic assessment	Average hair diameter and hair density	Terminal hair density
Secondary outcome	Terminal hair density, global photographic evaluation, quality-of-life evaluation	Safety of treatment	NA	Change in total hair density, and photographic evaluation

AGA, androgenetic alopecia; NA, not available; OM, oral minoxidil; RCT, randomized controlled trial; TM, topical minoxidil.

### Included study outcomes and conclusions

Hair density, terminal hair density, hair count, global photographic assessment, negative hair pull test and patient satisfaction outcomes were studied and are shown in Table 2. According to Ramos et al.,^12^ the mean difference in hair density in the OM group was greater than the mean difference in the TM group. In the study by Vahabi-Amlashi et al.^14^, almost no difference in the mean difference in hair density was observed between the OM group and the TM groups. The results from the study of Pehna et al. showed that there was not much difference in the mean difference in hair density between the OM group and the TM group.^15^ Ramos et al. observed that the mean difference in terminal hair density in the OM group was more than that observed in the TM group.^12^ Penha et al. observed that the mean difference in terminal hair density in the OM group was more than double that observed in the TM group.^15^ Asilian et al. observed telogen effluvium in 9% of the patients who received OM and in 13% of patients receiving TM.^13^ Penha et al. observed hypertrichosis in 66.7% of patients receiving OM, while hypertrichosis was seen in 33.3% of patients receiving TM.^15^

**Table 2:** Included study outcomes and conclusions

Study outcome	Ramos et al.^12^	Asilian et al.^13^	Vahabi-Amlashi et al.^14^	Penha 2024^15^
Hair density in the OM group (cm2)	164.6 (48.1) at baseline and 184.7 (57.1) at 6 months	NA	102.0 (79.2) at baseline and 109.8 (79.2) at 6 months	190.2 (61.2) at baseline and 201.0 (61.4) at 6 months
Mean difference in hair density in OM group	20.1	NA	7.8	10.8
Hair density in the TM group (cm2)	163.2 (46.0) at baseline and 176.3 (61.5) at 6 months	NA	107.4 (21) at baseline and 115.26 (133.2) at 6 months	207.4 (63.7) at baseline and 215.5 (58.9) at 6 months
Mean difference in hair density in OM group	13.1	NA	7.9	8.1
Terminal hair density in OM group (cm2)	106.5 (34.2) at baseline and 112.6 (36.4) at 6 months	NA	NA	91.2 (47.6) at baseline and 101.5 (54.4) at 6 months
Mean difference in terminal hair density in OM group	6.1	NA	NA	10.3
Terminal hair density in TM group (cm2)	113.3 (41.1) at baseline and 116.8 (44.9) at 6 months	NA	NA	105.3 (42.2) at baseline and 109.7 (49.0) at 6 months
Mean difference in terminal hair density in TM group	3.5	NA	NA	4.4
Hair count in OM group (mean)	Mean change from baseline was 17.68	NA	NA	NA
Hair count in TM group (mean)	Mean change from baseline was 25.27	NA	NA	NA
Improved on global photographic assessment in OM group, *n* (%)	18 (70)	NA	NA	20 (60.6)
Improved on global photographic assessment in OM group, *n* (%)	12 (46)	NA	NA	17 (48)
Patient satisfaction in OM group (%)	NA	91.4	NA	NA
Patient satisfaction in TM group (%)	NA	54.2	NA	NA
Negative hair pull test in OM group, *n* (%)	NA	77%	33 (91.7)	NA
Negative hair pull test in the TM group, *n* (%)	NA	40%	25 (69.4)	NA
Adverse effects in OM group	Pretibial oedema in 4%	Orthostatic hypotension in 3%, telogen effluvium in 9%, hypertrichosis in 6%	Hirsutism in 5.5%	Hypertrichosis in 66.7% of patients, headache in 18.2% of patients, itching on the scalp in 3%, nightmares in 3%, scalp eczema in 3%, shedding in 3%, insomnia in 3%, lower limb oedema in 3%, abdominal pain in 3%, lower limb pain in 3%
Adverse effects in TM group	Scalp pruritus in 19%	Telogen effluvium in 13.79%	Hirsutism in 6%, gut intolerance in 6%, and hypotension in 3%	Hypertrichosis in 33.3%, headache in 3%, itching on the scalp in 15.2%, nightmares in 6.1%, scalp eczema in 21.2%, shedding in 21.2%, insomnia in 6.1%, appetite increase in 3%
Conclusion	With a well-tolerated adverse effect profile and a safe profile, OM offers similar improvement as TM	OM may be as effective and safe as TM in AGA	OM is an effective new treatment for AGA and can be used as an alternative treatment to TM	OM did not demonstrate superiority over TM in treating AGA

Data are presented as mean (SD), unless otherwise stated. AGA, androgenetic alopecia; NA, not available; OM, oral minoxidil; TM, topical minoxidil.

### Hair density outcomes analysis (OM vs. TM)

**Figure 2:**
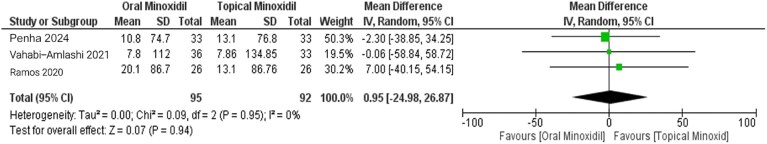
Forest plot showing a comparison between oral minoxidil and topical minoxidil for hair density outcome. CI, confidence interval.

Three studies investigated hair density in OM and TM treatment groups. A forest plot was constructed (Figure 2) to see the overall effect size in terms of hair density in these three studies. The results showed that there was no significant difference between OM and TM in terms of improving the hair density of these patients (overall mean difference 0.95, 95%CI −24.98 to 26.87).

### Risk of bias assessment

**Figure 3:**
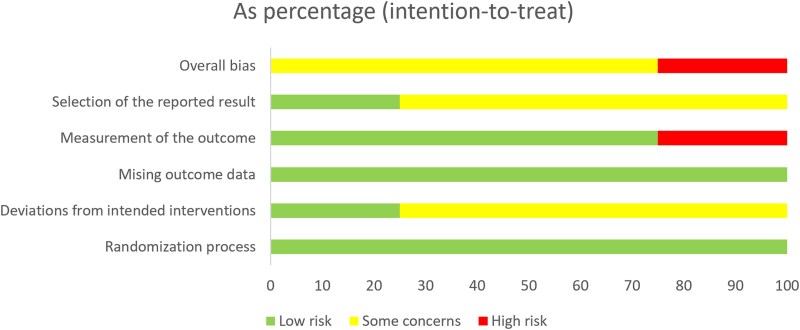
Risk of bias graph of the included studies.

**Figure 4:**
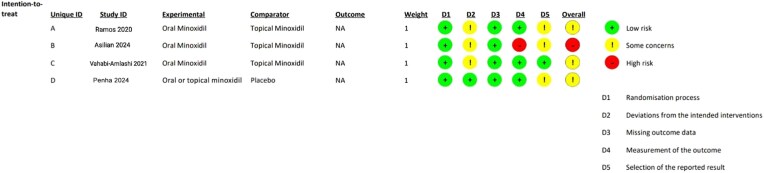
Risk of bias summary of the included studies.

Figures 3 and 4 show the risk of bias graph and risk of bias summary, respectively, of the included studies.

## Discussion

Comparing traditional and FDA-approved treatment modalities with newer treatment options is crucial when it comes to managing patients with AGA. TM is an FDA-approved drug, whereas OM is still not FDA-approved for use in AGA. OM, being a potentially newer treatment modality, has to be compared with TM for the treatment of AGA to assess whether OM can offer better treatment outcomes and a safer adverse effect profile than TM or not. Choosing the right treatments for AGA can be difficult, even though there are many medicinal, surgical, light-based and nutritional approaches available to halt or reverse the development of the condition.^7^

This study compared OM with TM in terms of various outcomes that were originally studied in the four included clinical trials. There was no significant difference between OM and TM when it came to improving hair density in patients with AGA. Only this outcome was statistically analysed because no other outcome was studied in all four, or in at least three of the four, included clinical trials. Hair diameter is another outcome that was studied; however, there was no difference between OM and TM in improving hair diameter in patients with AGA. The typical 5% topical solution was not more effective in increasing mean hair diameter than 1 mg OM taken once daily.^13^ One study showed that OM was better than TM in terms of improving the Sinclair hair-shedding score. The study showed that when it came to the Sinclair hair loss score, OM performed better than TM.^12^

As per the results of this study, hair density and diameter changed in both groups when compared to baseline values; however, the pattern of changes did not differ significantly between the two groups. This was shown in a clinical trial that compared the efficacy of oral TM in treating AGA.^14^

As far as terminal hair density is concerned, only two of the included studies investigated it in their original trials; therefore, this outcome could not be analysed.^12,15^ In both the studies that investigated terminal hair density, OM was found to be superior to TM, but this could not be proved statistically because of the lack of data from any other included clinical trial; we need more clinical trials that can study and compare the effects of OM vs. TM when it comes to improving terminal hair density.

Thus, OM was not found to be superior to TM in any of the four included clinical trials or in pooled analysis of hair density outcome. OM can be used as an adjunct to TM or as a second-line treatment option. OM is an effective new treatment for AGA and can be used as an alternative treatment to TM. For individuals with AGA who do not comply well with TM or who are intolerant to it, OM may be a viable choice. Another treatment modality known as platelet-rich plasma (PRP) is also available for treating AGA. However, there are certain issues with the literature available on PRP as it reports various PRP methods and lacks consistent agreement that would enable objective analytical comparison. Clinical reports of PRP preparation procedures varies greatly, and most lack enough details to enable replication of the procedure. Furthermore, it is not possible to compare the PRP products that are being given to patients due to the present reporting of PRP preparation and composition. For repeatability and study comparison, a thorough, accurate and step-by-step explanation of the PRP preparation procedure is necessary.^16^

Notably, the OM dose of 1 mg daily administered in the included clinical studies may have a substantial impact on the outcomes. This dosage is quite modest, especially for men, who frequently use amounts of up to 5 mg daily. In the same way, OM up to 2.5 mg daily is usually well tolerated and thought to be more beneficial for women. Therefore, future clinical trials might include the use of OM at a dosage of 5 mg daily and compare it with other approved drugs used for AGA. Thus, OM 5 mg daily could prove to be more useful in terms of improving outcomes in patients who are treated for AGA. A truer comparison between OM and TM can be drawn by using OM 5 mg daily rather than 1 mg daily. These subjects warrant more clinical trials so that OM and TM can be better compared. At the same time, the outcomes must be kept uniform among the clinical trials so that a pooled analysis of outcomes can be done. Only then can the two drugs be better compared and a more appropriate comparison drawn, which could be a useful source of evidence-based practice. However, the current review demonstrates that the efficacy of OM is comparable to that of TM in treating AGA, and thus OM could be of help in treating patients with AGA provided OM can gain FDA approval so that it can be used in practice.

The efficacy of OM is comparable to TM in terms of improving outcomes such as hair density and terminal hair density in patients with AGA. Hypertrichosis is found to be more extensive in patients who receive OM. OM can be used as an adjunct or as a second-line treatment option for AGA. The comparability of OM and TM in terms of improving outcomes in AGA warrants the need for more clinical trials on this subject which may lead to FDA approval for OM.

## Data Availability

The data relevant and related to this study are available in Zenodo at https://zenodo.org/records/13893013, and can be accessed at: https://doi.org/10.5281/zenodo.13893013.^17^
